# Regulatory networks of circRNAs related to transcription factors in *Populus euphratica* Oliv. heteromorphic leaves

**DOI:** 10.1042/BSR20190540

**Published:** 2019-12-13

**Authors:** Lianghong Bao, Shaowei Qin, CaiLin Li, Zhongzhong Guo, Lifeng Zhao

**Affiliations:** 1 College of Life Sciences, Tarim University, Alar 843300, China; 2 Key Laboratory of Protection and Utilization of Biological Resources in Tarim Basin, Tarim University, Alar 843300, China

**Keywords:** Populus euphratica Oliv., circRNAs, regulatory networks, transcription factors

## Abstract

Circular RNAs (circRNAs) are a novel class of non-coding RNAs that are characterized by a covalently closed circular structure. They have been widely found in *Populus euphratica* Oliv. heteromorphic leaves (*P.* hl). To study the role of circRNAs related to transcription factors (TFs) in the morphogenesis of *P.* hl, the expression profiles of circRNAs in linear, lanceolate, ovate, and broad-ovate leaves of *P. euphratica* were elucidated by strand-specific sequencing. We identified and characterized 22 circRNAs related to TFs in *P.* hl at the four developmental stages. Using the competing endogenous RNAs hypothesis as a guide, we constructed circRNA–miRNA–TF mRNA regulatory networks, which indicated that circRNAs antagonized microRNAs (miRNAs), thereby influencing the expression of the miRNA target genes and playing a significant role in transcriptional regulation. Gene ontology annotation of the target TF genes predicted that these circRNAs were associated mainly with the regulation of leaf development, leaf morphogenesis, signal transduction, and response to abiotic stress*.* These findings implied that the circRNAs affected the size and number of cells in *P.* hl by regulating the expression of TF mRNAs. Our results provide a basis for further studies of leaf development in poplar trees.

## Introduction

*Populus euphratica* Oliv*.* is a typical stress-resistant tree that grows in desert areas of China. Its stress-resistant characteristics include tolerance to salt, alkali, drought, and cold [1]. The young leaves of *P. euphratica* are linear and have a leaf index (LI, leaf length/leaf width) ≥5. As the trees age, the leaves gradually become lanceolate (La, 2≤LI<5), ovate (Ov, 1≤LI<2), then broad-ovate (Bo, LI<1). Therefore, *P. euphratica* has typical heteromorphic leaves, which make it an ideal model for studying leaf morphogenesis in plants [2]. Transcription factors (TFs) are known to play crucial roles in the growth and development of plants by regulating the expression levels of genes [3]. For example, previous reports found that programmed cell death was regulated by basic-helix-loop-helix (bHLH) TFs in *Arabidopsis* endosperm [4] and that AP2/ERF TFs could regulate leaf development in *Arabidopsis* [5].

Non-coding RNAs can indirectly regulate the development of plants [6]. Circular RNAs (circRNAs) are endogenous non-coding RNAs that have a covalently closed circular structure, which makes them more stable than linear RNAs [7]. CircRNAs act as microRNA (miRNA) sponges and circRNA–miRNA interactions influence gene expression and play vital roles in plant and animal development [8–10]. For example, Os08circ16564 was predicted to harbor the target sites of miR172 and miR810 in *Oryza sativa* L. [11]. Databases of circRNAs have recently become available, including CircNet [12] and PlantcircRNABase (http://ibi.zju.edu.cn/plantcircbase), which has allowed the regulatory roles of predicted circRNA–miRNA–TF mRNA networks to be further investigated. Salmena et al. proposed a competing endogenous RNA (ceRNA) hypothesis that ceRNAs interact using miRNA response elements (MREs) to form transcriptome-wide regulatory networks [13]. Some circRNAs located in the nucleus of cells that harbor miRNA binding MREs form circRNA–miRNA axes that regulate gene expression at the transcriptional or post-transcriptional level [14–16]. For example, circRNAs were found to function as biomarkers of the homeotic MADS-box TF family in *Arabidopsis* [17]. It has been suggested that most circRNAs play regulatory roles at the transcriptional or post-transcriptional levels [18]. Li et al. [19] identified circRNAs that were involved in the morphogenesis of *Populus euphratica* Oliv. heteromorphic leaves (*P.* hl), and Wang et al. [20] found that circRNA interacted with miRNA156 to regulate the cognate linear transcripts of TRF-1 in *Phyllostachys edulis*. However, there are few studies about the regulation of TFs by circRNAs at the transcriptional or post-transcriptional level in *P.* hl.

In the present study, we analyzed the expression profiles of circRNAs in *P.* hl at four developmental stages (Bo, Ov, La, and Li) by strand-specific sequencing, small RNA sequencing (RNA-seq), and bioinformatic tools [21]. We identified 258 miRNAs in *P.* hl, 167 were known annotated miRNAs and 91 were novel miRNAs. Among them, 52 miRNAs were differentially expressed (P <0.05). The RNA-seq data analysis detected 8942 mRNAs that were differentially expressed among the *P.* hl at different developmental stages. A total of 1149 circRNAs were predicted from the RNA-seq data [22]. Combined with the ceRNA hypothesis, we constructed circRNA–miRNA–TF mRNA networks to predict the role of circRNAs in *P*. hl.

## Materials and methods

### Plant materials and RNA isolation

We collected *P. euphratica* leaves at four developmental stages from trees growing in the Tarim Basin, Xinjiang (81°17 poplar 56.52" E; 40°32 poplar 36.90" N). Li, La, Ov, and Bo leaves were selected as materials when there are 7–13 leaves on a bud. Each sample was biologically repeated three times. The Li, La, Ov, and Bo samples were named Li1, Li2, and Li3; La1, La2, and La3; Ov1, Ov2, and Ov3; and Bo1, Bo2, and Bo3. The samples were frozen immediately in liquid nitrogen and stored at −80**°**C before processing. Total RNA was extracted using a mirVanamiRNA isolation kit (Ambion) according to the manufacturer’s instructions, and total RNA concentration and quality were measured using an Agilent 2100 Bioanalyzer (Agilent Technologies, Santa Clara, CA, U.S.A.). The strand-specific sequencing process was performed as described by Levin et al. [21]. All the libraries were built from high-quality RNA samples with 28S/18S >1 and A260/A280 between 1.8 and 2.1.

### Identification of differentially TF mRNAs in *P.* hl

The clean reads were aligned with sequences in the plant transcription factor database (PlnTFDB; http://plntfdb.bio.uni-potsdam.de/v3.0/) to identify candidate TFs and the relative abundance of the transcripts was calculated using Bowtie2 [23] and eXpress [24]. The expression levels of the TF mRNAs were quantified as fragments per kb per million reads (FPKM) [25]. Fold changes (FCs) in the expression levels of genes between two libraries were calculated as log2 ratios, and the data were normalized using DESeq2 [26]. TFs with logFC ≥ 2 or ≤−2 and *P* < 0.05 were considered to be differentially expressed.

### Identification of differentially expressed circRNAs in *P.* hl

According to the data obtained from 12 samples sequenced, circRNAs were identified using CIRI software [27], circRNAs with *P* < 0.05 and logFC ≥ 2.0 or ≤ −2.0 were considered to be differentially expressed. The expression levels of the circRNAs in each library were quantified as spliced reads per million (RPM), differential expression analysis and identification of circRNA were the same as for the TFs.

### Bioinformatics identification of miRNAs in *P.* hl

The small RNA was isolated and purified from the total RNA and used as sequencing sample. The miRNAs were sequenced by Illumina high-throughput sequencing, and small RNA reads were obtained by Illumina analysis (OE Biotechnology, Shanghai, China) and compared with sequences in miRBase v.21 (http://www.mirbase.org/) [28] to identify known miRNAs. The small RNAs that had no matches in miRBase were analyzed using miRDeep2 [29] to detect novel miRNAs. Differential expression analysis and identification of miRNAs were the same as for the TFs.

### Functional predictions of RNAs

The sequences of the identified TFs in the *P.* hl were compared with the *Arabidopsis* transcriptome sequence (GCF_000001735.3_TAIR10_rna.fna.gz; https://www.ncbi.nlm.nih.gov/genome/?term = arabidopsis+thaliana) to obtain the homologous genes of these TFs in *Arabidopsis*. The Gene Ontology (GO) annotations [30] of the homologous genes were used to assign functions to the TFs. Then, the GO enrichment was performed with Omicshare (https://www.omicshare.com/tools/Home/Report/goenrich). The functions of the miRNAs and circRNAs were predicted from the functions of their target TFs.

### Interaction analysis between different RNAs and network construction

We used the psRNATarget server (http://plantgrn.noble.org/psRNATarget/) to predict the target mRNAs of the identified miRNAs and circRNAs [22]. Then candidate miRNA–mRNA pairs were identified according to their MREs and by negative correlation of their expression profiles and circRNA–mRNA pairs were identified according to same MREs and positive correlation of their expression profile. Cytoscape software [31] was used to construct the regulation networks. Simultaneously, a heat map of differential expressed circRNAs related to TF mRNAs was built using a web tool from Omicshare (https://www.omicshare.com/tools/Home/Soft/heatmap).

### Validation by quantitative RT-PCR (qPCR)

To validate the reliability of the sequencing results, a line chart was built using EXCEL software. Total RNA was extracted from the Li, La, Ov, and Bo samples using Trizol reagent (Invitrogen, CA, U.S.A.). The qPCRs were performed using a qPCR kit and TB Green™ Premix Ex Taq™ (Takara, Dalian, China). The expression profiles of nine randomly selected RNAs, namely three TFs (XM-011016290.1, XM-011022483.1, and XM-011016291.1), three circRNAs (circRNA-0979, circRNA-1102, and circRNA-0168), and three miRNAs (NW-011500067.1, ptc-miR473a, ptc-miR160e), were verified by qPCR. The TF mRNAs and circRNAs were reverse transcribed using random primers, and the miRNAs were reverse transcribed using specific primers. A Tiangen kit was used for miRNA detection, and a Takara kit was used for circRNA and mRNA detection. Primers were designed by primer-blast in NCBI (www.ncbi.nlm.nih.gov/tools/primer-blast/), and 18S RNA was used as the internal reference. All the PCR primers are listed in Supplementary Table S1. Quantification of RNA expression (circRNAs, miRNAs, and TF mRNAs) was performed using the comparative Ct method. Fold changes (FCs) in the expression levels of RNAs were normalized as the ratio with 18S. Three technical replicates and three biological replicates were performed for each RNA sample.

### Validation of regulatory relationships of circRNAs related to TFs in *P.* hl

Sixteen *P. euphratica* leaf samples (Li1, Li2, Li3, and Li4; La1, La2, La3, and La4; Ov1, Ov2, Ov3, and Ov4; and Bo1, Bo2, Bo3, and Bo3) were combined in liquid nitrogen. Total RNA (pooled sample) was isolated using Trizol reagent (Invitrogen) and treated with DNase I (Takara) and RNase R (Geneseed Biotech Co.). Genomic DNA (gDNA) was extracted from the pooled sample using a MiniBEST Plant Genomic DNA Extraction Kit (Takara). Divergent primers were designed to cross the head-to-tail splicing junction using PRAPI [32], and convergent primers were designed as positive controls. Six circRNAs were selected randomly to validate their junctions in *P*. hl. The RNA was reverse-transcribed to cDNA using the PrimeScript™ II 1st Strand cDNA Synthesis Kit (Takara) with 1 μg total RNA and random 6-mers. The selected circRNAs were amplified and PCR products with the predicted sizes were visualized on a 2% agarose gel stained with ethidium bromide. All the primers are listed in Supplementary Table S2.

We identified miRNAs and TF mRNAs that may have regulatory relationships with six randomly selected circRNAs in the 16 samples of *P.* hl. The expression profile of the RNAs with possible circRNA–miRNA–TF mRNA regulatory relationships was verified by qPCR (the method referred to 2.7 section). All the PCR primers are listed in Supplementary Table S3. The correlation coefficients between the circRNA, miRNA, and TF mRNA expression profiles were calculated using Pearson coefficients with a web tool from Omicshare (https://www.omicshare.com/tools/Home/Soft/ica).

## Results

### Identification of differentially expressed circRNAs related to TFs in *P.* hl

**Figure 1:**
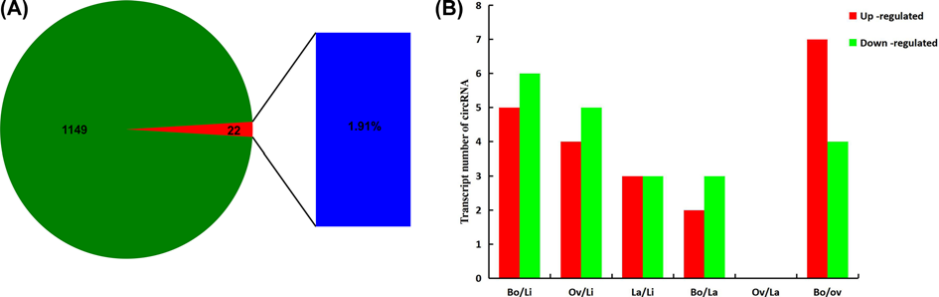
Identification of circRNAs related to TFs in *P. euphratica* heteromorphic leaves Proportion of circRNAs related to TFs (**A**). Differentially expressed circRNAs in the pairwise comparisons (**B**). Linear leaves, lanceolate leaves, ovate leaves, and broad-ovate leaves defined as Li, La, Ov, and Bo, respectively.

A total of 1149 circRNAs were identified in *P.* hl by strand-specific RNA sequencing and small RNA sequencing. Among them, 22 (1.91%) circRNAs related to TFs were predicted from the relationships in the circRNA–miRNA–TF mRNA regulatory networks using psRNATarget (http://plantgrn.noble.org/psRNATarget/) (Figure 1A). Notably, the numbers of up-regulated circRNAs were highest in Li (youngest) and Bo (oldest) leaves and lowest in the Ov leaves. Five circRNAs were up-regulated and six were down-regulated in the Bo/Li comparison, and no differentially expressed circRNAs were found in the Ov/La comparison (Figure 1B).

### Expression profiles of circRNAs related to TFs in *P.* hl

**Figure 2:**
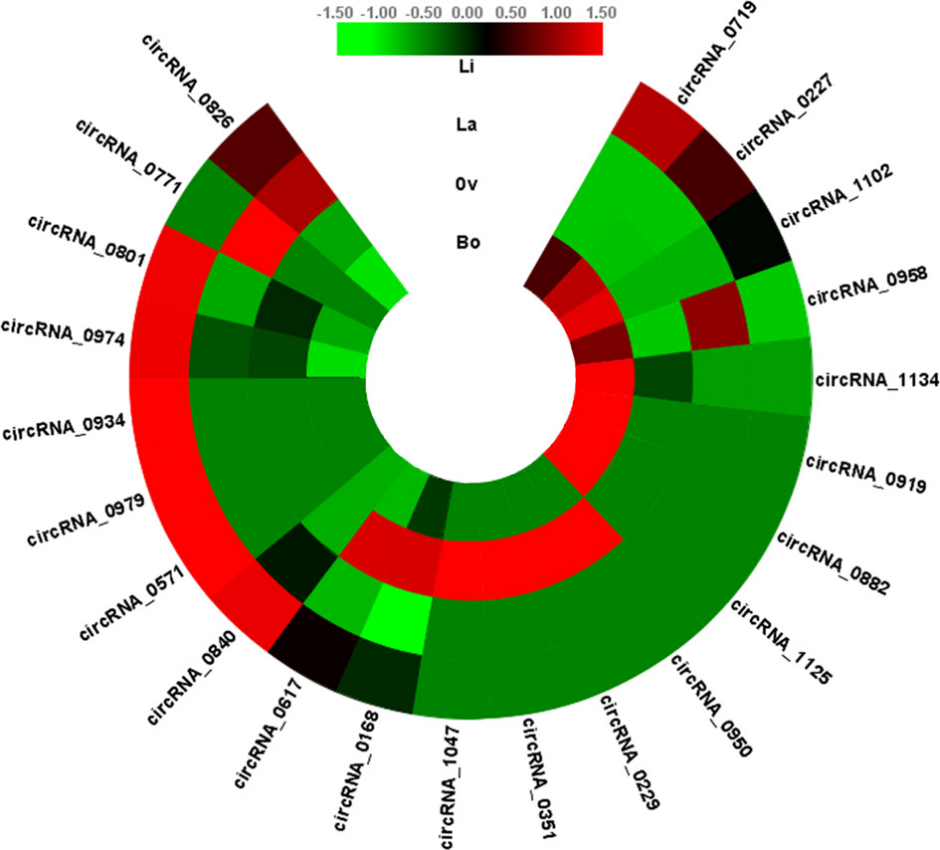
Heat map of the expression profiles of circRNAs was built among four leaf shapes in *P.* hl by omicshare FPKM values were calculated from the RNA-seq data and normalized by log2 transformation. The color scale indicates the log2 transformed values; green indicates low expression, red indicates high expression.

We investigated the transcript levels of the circRNAs in *P.* hl by analyzing the RNA-seq transcriptome data. A heat map of the circRNA expression profiles (RPM values) among the four leaf shapes (Li, La, Ov, Bo) showed that the 22 circRNAs related to TFs were widely expressed in *P.* hl (Figure 2). We found that the differences in expression levels were most significant in the Li and Bo leaves. The diversity of expression profiles indicated that the circRNA may have different functions in the leaves at different developmental stages.

### GO enrichment of TFs targeted by circRNAs in *P.* hl

**Figure 3:**
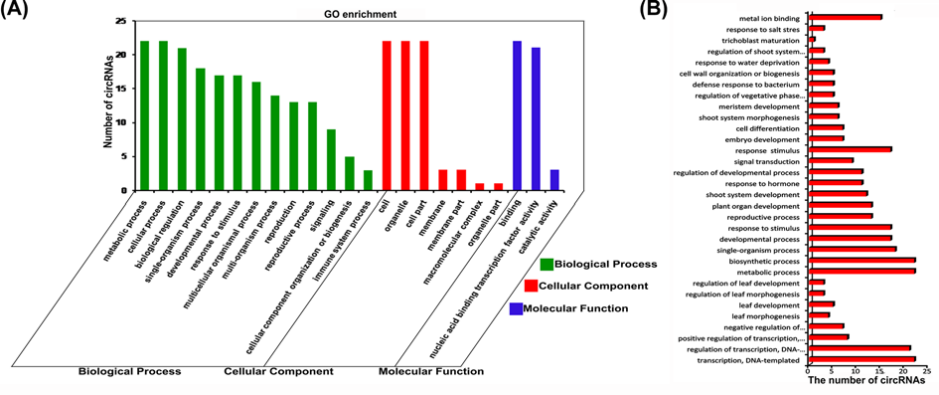
Distribution of GO terms and number of circRNAs that targeted the annotated TFs GO annotations under the three main GO categories (**A**). GO annotations under biological function (**B**). The gene set was enriched by omicshare, and a *P*-value of 0.05 was set for the gene set.

The functions of the TFs that were targets of the 22 circRNAs were predicted using GO (Figure 3A). Under biological process, the enriched terms included reproduction, metabolic process, signal transduction, developmental process, and stress response. Under molecular function, the enriched terms included binding to nucleotides and participating in transcriptional regulation. Five of the TFs targeted by circRNAs were associated with regulation of leaf shape development, four were associated with leaf morphogenesis, and three of them were predicted to regulate morphological development and formation of leaves (Figure 3B).

### Construction of circRNA–miRNA–TF mRNA regulatory networks

**Figure 4:**
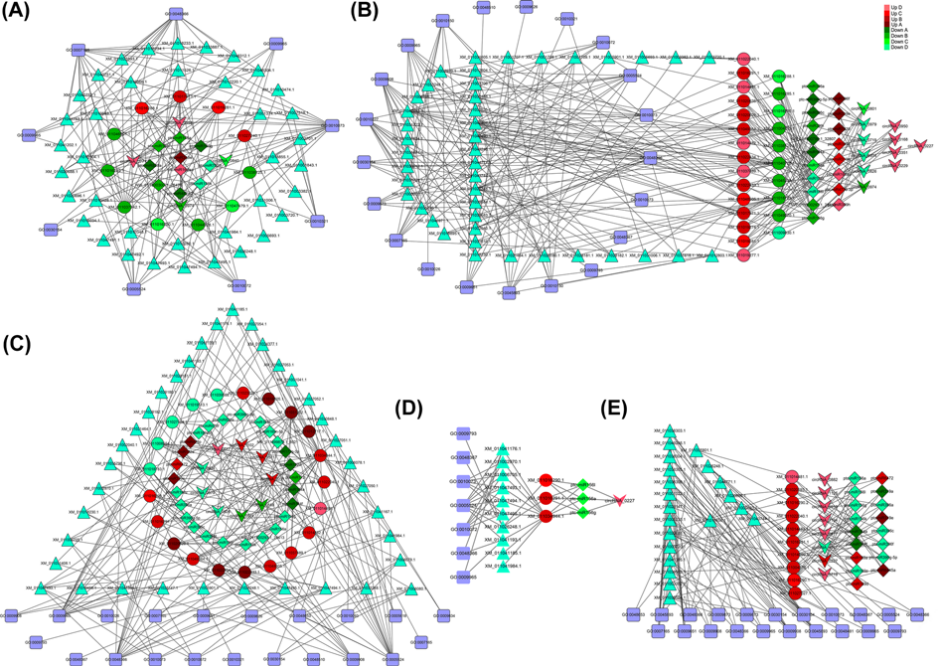
Regulatory circRNA–miRNA–TF mRNA networks in *P. euphratica* heteromorphic leaves The networks were constructed for differentially expressed circRNAs in the Li/La (**A**), Li/Ov (**B**), Li/Bo (**C**), La/Bo (**D**), and Bo/Ov (**E**) comparisons by cytoscape 3.6.0. V shapes that indicate circRNAs, diamonds indicate miRNAs, circles indicate TFs, triangles indicate target genes, and squares contain the GO IDs. The color scale indicates the regulatory relationships: red, up-regulated; green, down-regulated.

We used bioinformatics tools to analyze downstream genes regulated by the TFs targeted by the 22 circRNAs to predict the regulatory role of circRNAs that were differentially expressed among the *P.* hl at different developmental stages. We constructed regulatory networks that contained 22 circRNAs, 33 miRNAs, and 59 TFs. Four differentially expressed circRNAs in the La/Li comparison regulated the expression of 11 TF mRNAs by antagonizing 10 miRNAs. These TFs were predicted to regulate the expression of 39 downstream target genes. For example, circRNA-0974, which was differentially expressed in La/Li, affected the expression of the WER-like TF gene (XM-011046001.1) that regulated the expression of the genes encoding calcium-dependent protein kinase SK5 (XM-011001374.1). The GO annotations indicated that circRNA-0974 targeted TFs associated with developmental process (GO: 0005524) and cell differentiation (GO: 0030154) (Figure 4A).

In the Ov/Li comparison, 11 circRNAs, 19 miRNAs, and 23 TF mRNAs were included in the regulatory network. The 11 differentially expressed circRNAs regulated 42 downstream target genes (Figure 4B) that were involved mainly in signal transduction, trichomes differentiation, cell processes, transmission tissue development, leaf formation, leaf morphology, and leaf development. For example, circRNA-0719 antagonized three miRNAs (ptc-miR399i, ptc-miR399d, and ptc-miR399h), which down-regulated the expression of the HEC2-like TF gene (XM-011045233.1), a target of ptc-miR399. This affected the expression of the downstream target gene cyclin-P3-1-like (XM-011034408.1), which was predicted to be associated with leaf morphogenesis (GO: 0009965) and cellular process (GO: 0005524).

In the Bo/Li comparison, 9 circRNAs, 24 miRNAs, and 21 TF mRNAs were included in the regulatory network, and the 21 TFs regulated 37 downstream target genes (Figure 4C). For example, down-regulated circRNA-0934 up-regulated the expression of ptc-miR172, which down-regulated the expression of its target TF (XM-011008554.1). This affected the expression of the downstream target genes GTL1-like TFs (XM-011006078.1, XM-011006079.1, and XM-011006080.1) that were predicted to be involved in development of leaves and differentiation of shoot tips. These results suggest that one upstream TF can regulate multiple downstream target genes.

In the Bo/La comparison, up-regulated circRNA-0227 down-regulated the expression of ptc-miR156a, ptc-miR156g, and ptc-miR156l, which regulated the expression of three TFs (XM-011034944.1, XM-011016291.1, and XM-01106290.1). This affected the expression of 10 downstream target genes (Figure 4D) that were predicted to be involved in the maintenance of leaf meristem, which led to the transition of leaf polarity from the adaxial–abaxial axis to the medial–lateral axis direction.

In the Bo/Ov comparison, 8 circRNAs, 17 miRNAs, and 10 TFs were included in the regulatory network. The 10 TFs regulated 21 downstream target genes (Figure 4E). For example, down-regulated circRNA-0617 regulated the expression of two miRNAs (ptc-miR169a, ptc-miR169d), which down-regulated the expression of nuclear TF Y subunit (XM-011022040.1). This affected the expression of the downstream target genes peroxiredoxin-2F (XM-011003720.1), transcription elongation factor S-II-like (XM-011003382.1), and transcription factor IIIA (XM-011005693.1) that were predicted to participate in signal transduction, positively regulate transcription, and respond to biological processes related to salt stress.

### Verification of gene expression by qPCR

**Figure 5:**
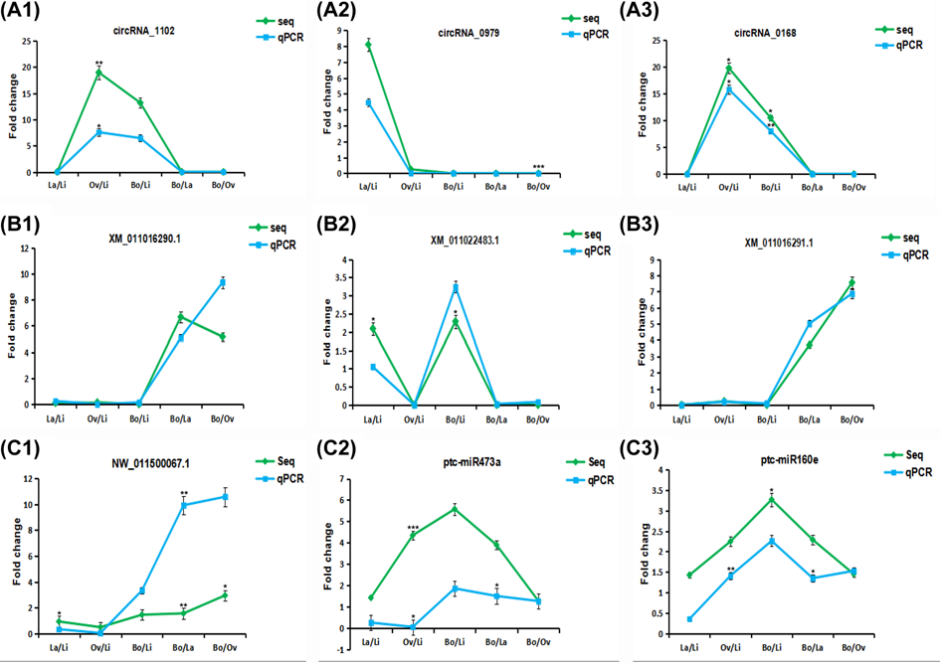
Validation of gene expression by qPCR CircRNAs (**A1–A3**), mRNAs (**B1–B3**), and miRNAs (**C1–C3**). *P < 0.05, **P < 0.01, and ***P < 0.001 are the significance values of the comparison of sample pairs obtained using DESeq2. *P < 0.05, **P < 0.01, and ***P < 0.001 are the significance values of the comparison of sample pairs obtained by Student’s test. Error bars indicate ± SD.

To verify the reliability of the strand-specific transcriptome sequencing results (Figure 5), we randomly selected 3 circRNAs, 3 mRNAs, and 3 miRNAs that were differentially expressed in *P.* hl and analyzed their expression by qPCR. The trends of the expression levels of these RNAs determined by qPCR were basically consistent with the expression levels obtained by transcriptome sequencing. This confirmed the reliability of the strand-specific sequencing results.

### Validation of regulatory relationships in *P*. hl

**Figure 6:**
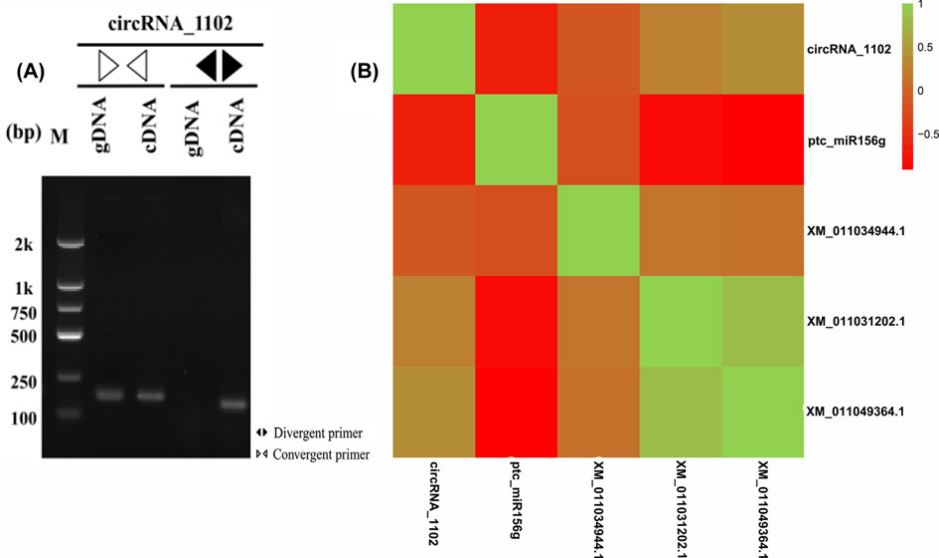
Regulatory relationship of a representative circRNA associated with TFs in *P. euphratica* heteromorphic leaves (**A**) Amplification products of circRNA_1102 using gDNA and cDNA (RNase R+) as templates with convergent and divergent primers separated on agarose gel. (**B**) Matrix of Pearson correlation coefficients (*r*) was constructed among circRNA, miRNA, and TF mRNAs using Omicshare. The color scale indicates the range of Pearson correlation coefficients.

To validate the regulatory relationship of the circRNAs associated with TFs in the *P*. hl, we used gDNA and cDNA (RNase R+) as templates with convergent and divergent primers. Five of six selected circRNAs related to TFs were amplified successfully in the cDNA sample (Supplementary Figure S1). The results for circRNA_1102 showed that the divergent primers successfully amplified only the cDNA, whereas the convergent primers successfully amplified gDNA and cDNA (Figure 6A). We calculated the Pearson correlation coefficient (*r*) among circRNAs, miRNAs, and the linear TF mRNAs in 16 *P*. hl samples. In total, 17 of 21 regulatory relationships had negative Pearson correlation coefficients between the expression levels of the miRNAs and circRNAs, and between the expression levels of the miRNAs and TF mRNAs as determined by qPCR; however, the remaining regulatory relationships showed positive correlation (Supplementary Tables S4 and S5). For example, the Pearson correlations between circRNA_1102 and ptc-miR156g and between ptc-miR156g and XM_011034944.1, XM_011031202.1, or XM_011049364.1 were negatively correlated (Figure 6B). These results suggest that miRNAs may be affected by multiple circRNAs and can affect the expression of their target TFs, and that, conversely circRNA_1102 could be regulated by ptc-miR156.

## Discussion

Some genes in plant leaves determine leaf shape by regulating leaf developmental morphology and metabolism, and participate in the polarity of the adaxial–abaxial, apical–basal, and medial–lateral axes [2]. Leaf shape development is regulated by strict genetic mechanisms [33]. The important regulatory roles between circRNAs and TFs have been confirmed in animals. For example, Ma et al. [34] confirmed that circMAN2B2 inhibited miR-1275 to promote the expression of the FOXK1 TF in human lung cancer tissue. The model plant *P. euphratica* has heteromorphic leaves, but the molecular regulation mechanism of circRNAs related to TFs in the morphogenesis of *P*. hl was unknown. Therefore, in the present study we investigated the regulation mechanism of circRNAs on the morphogenesis of *P.* hl at four developmental stages, and found that 22 differentially expressed circRNAs related to TFs that participated in the morphogenesis of *P*. hl (Figure 1A).

Previous studies found the expression of circRNAs were tissue and organ-specific [35,36]. We found that the expression of circRNAs was more spatio-temporal specificity than the expression of mRNAs. The differences of circRNA expression patterns in the different leaves of *P. euphratica* (Li, La, Ov, Bo) indicated there was significant spatio-temporal specificity of circRNAs in the morphological development of *P.* hl (Figure 2), implying that circRNAs might have a cumulative effect as the leaf shape develops from Li to Bo.

From the RNA-Seq results and qPCR results, it was found that the expression patterns of most miRNAs were opposite to those of the circRNAs and TF mRNAs, suggesting that circular RNAs might act as miRNA sponges and could also be degraded by miRNAs in the *P*. hl (Figure 6B; Supplementary Tables S4 and S5). However, the qPCR results also revealed a few RNAs had positive correlations, indicating the process and the mechanism might be more complicated than we have known. We also found that many circRNAs were involved in leaf development of *P. euphratica* CircRNA-0974, circRNA-0719, circRNA-0934; circRNA-0227 and circRNA-0617 were predicted to play important roles in leaf shape changes. Among them, circRNA-0719 was differentially expressed in all the pairwise comparisons and the highest expression among the four leaf types was in the Li leaves (Figure 2). The results indicated that circRNA-0719 may affect the downstream target gene cyclin-P3-1-like (XM-011034408.1) to influence the polarity in the apical–basal axis direction by indirectly regulating the expression of the HEC2-like TF gene (XM-011045233.1), which is homologous to bHLH TF genes (Figure 4B). MacAlister et al. [37] found that the bHLH TF family gene ICE1 affected leaf morphology by interacting with MUTE and FAMA, which is generally consistent with our results. CircRNA-0934 regulated the expression of the floral homeotic protein APETALA 2-like TF gene (XM-011008554.1), a homolog of the AP2 TF, and participated in the development of leaf morphology (Figure 4C). Morcillo et al. [38] showed that the AP2 TF in oil palm was related to early development of leaf primordium. CircRNA-0227 was highly expressed in the Bo leaves and was predicted to regulate the expression of a squamosa promoter-binding-like TF (XM-011034944.1), a homolog of the SPL TF gene, by interacting with miR156, thereby affecting leaf meristem and regulating the growth of leaves toward the medial–lateral axis (Figure 4D). Furthermore, miR156 was down-regulated in *P.* hl, indicating that miR156 might play a vital role in the development of *P*. hl. SPL was found to be regulated by miR156 and was expressed mainly in juvenile shoots and leaves in rice [39]. The up-regulated expression of circRNA-0617 in the Bo/Ov comparison affected the expression of the nuclear TF Y subunit (XM-011022040.1), a homolog of the NF-YA TF gene, by interacting with miRNA169 (Figure 4E). The GO annotations indicated that XM-011022040.1 might be involved in signal transduction and response to salt stress in *P.* hl, implying that circRNA_0617 might participate in the abiotic stress response to promote the development of the medial–lateral axis or inhibit the apical–basal axis.

## Conclusions

The circRNA family of regulatory RNAs participates in a variety of biological functions. In the present study, we analyzed the expression profile of circRNAs related to TFs by strand-specific RNA sequencing of *P.* hl at four developmental stages*.* We identified 22 differentially expressed circRNAs related to TFs and established circRNA–miRNA–TF mRNA regulatory networks. Using the ceRNA hypothesis as a guide, we predicted that these circRNAs regulated the expression of 59 TFs by antagonizing 33 miRNAs. These interactions may play significant roles in regulating leaf development, leaf morphology, signal transduction, and response to abiotic stress in *P.* hl. Therefore, we propose that circRNAs may be involved in changing the shape and size of *P.* hl by regulating the expression of TF genes. Our results provide a foundation for further studies of the molecular mechanisms of leaf morphogenesis in *P. euphratica*.

## Supplementary Material

Supplementary Figure S1 and Tables S1-S5

## Abbreviations

ceRNA: competing endogenous RNA
circRNA: circular RNA
FC: fold change
gDNA: genomic DNA
MRE: miRNA response elements
RPM: reads per million
TF: transcription factor
