# Supplementary material for: Regulatory networks of circRNAs related to transcription factors in Populus euphratica Oliv. heteromorphic leaves
Source: Biosci Rep. 2019 Dec 13;39(12):BSR20190540. doi: 10.1042/BSR20190540 (PMC6911160; doi:10.1042/BSR20190540)
Supplement: Supplementary Figure S1 and Tables S1-S5 [file BSR-2019-0540_supp.pdf]

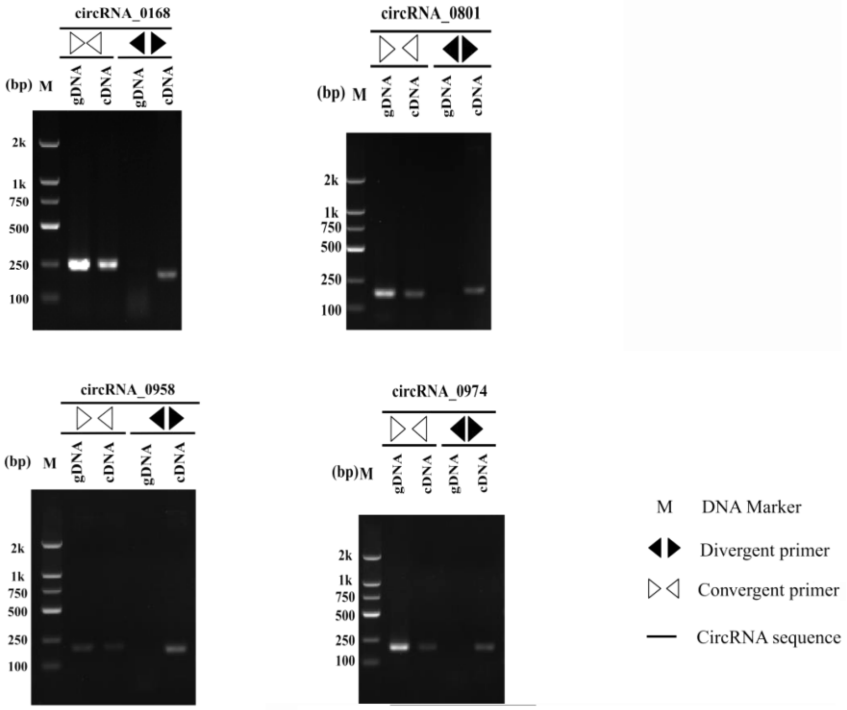

Fig. S1. CircRNAs related to TFs that were successfully amplified in *P*.hl .

Table S1 Sequences of primers were used in qPCR.

RNAs*	Primers	Sequence (5'-3')	Product	Note
18s RNA	Forward	GTGGTGGTGCATGGCCGTTC	80	mRNA
18s RNA	Reverse	TAGCAGGCTGAGGTCTCGTTCG		
circRNA_0168	Forward	AGGCCGAATCATTGGGTTTGA	196	circRNA
circRNA_0168	Reverse	ACCCCAAGCTGTTCCACTGA		
circRNA_0979	Forward	CAGTTCTGAATTTTGCAGCCGC	171	circRNA
circRNA_0979	Reverse	GCAGCAACTGACCGCTACAG		
circRNA_1102	Forward	TGGAGGTGCGTCAATGTCCA	144	circRNA
circRNA_1102	Reverse	TCCACGTTCTACAAGTGCTGGT		
XM_011016290.1	Forward	CTCTCACGGCTGTTGCTGAG	182	mRNA
XM_011016290.1	Reverse	TTTCCTGTGAGGCGGTTTCC		
XM_011016291.1	Forward	GTTGCCAGGCAGGACGGATAAC	161	mRNA
XM_011016291.1	Reverse	TGGATGGTGAAGACGGCAATGATG		
XM_011022483.1	Forward	TAAGCCATTGCCATCTCCACAACC		
XM_011022483.1	Reverse	GGCGTGCCTCTACCAGAATTGTC	121	mRNA
NW_011500067.1	Forward	cgaggcggaTgTagccaagTgga		miRNA
ptc-miR473a-3p	Forward	TGAGGCCTTTGGGGGAGAGTGG		miRNA
ptc-miR160e-5p	Forward	TGCCTGGCTCCCTGAATGCCA		miRNA

RNAs* include mRNA, miRNA and circRNA.

Table S2 Randomly selected primers of circRNAs in *Populus euphratica* Oliv. heteromorphic leaves

circRNAs	Primers	Sequence (5'-3')	product
circRNA_1102	Forward	GGAGGTGCGTCAATGTCCAA	216
circRNA_1102	Reverse	TCCACTGTAAAATACTTGCTGCCA	
circRNA_0168	Forward	ACTTGAGGATTGAAGGCCGAA	213
circRNA_0168	Reverse	TGATACCCCAAGCTGTTCCAC	
circRNA_0958	Forward	GGGGTGGTAGTTGCTGTGAT	230
circRNA_0958	Reverse	ACAGGCATCAGCCATCACTTG	
circRNA_0801	Forward	TGAGCCAAGCAGTCTATGCG	216
circRNA_0801	Reverse	TTTGTCTTGCGGCTGGTTGG	
circRNA_0227	Forward	AATTGGTGGTGCCCTTCCAC	222
circRNA_0227	Reverse	AGCCTTTCCTTCTTCGCAACC	
circRNA_0974	Forward	TGACAAGAAATGACTCTCACATGCT	210
circRNA_0974	Reverse	GTATGTTGGACGGCATACATCCT	

Table S3 Randomly selected primers of RNAs in circRNA-miRNA-TF mRNA regulatory networks

RNAs*	Primers	Sequence (5'-3')	Product	Note
circRNA_1102	Forward	GGAGGTGCGTCAATGTCCAA	216	circRNA
circRNA_1102	Reverse	TCCACTGTAAAATACTTGCTGCCA		
circRNA_0168	Forward	ACTTGAGGATTGAAGGCCGAA	213	circRNA
circRNA_0168	Reverse	TGATACCCCAAGCTGTTCCAC		
circRNA_0958	Forward	GGGGTGGTAGTTGCTGTGAT	230	circRNA
circRNA_0958	Reverse	ACAGGCATCAGCCATCACTTG		
circRNA_0801	Forward	TGAGCCAAGCAGTCTATGCG	216	circRNA
circRNA_0801	Reverse	TTTGTCTTGCGGCTGGTTGG		
circRNA_0227	Forward	AATTGGTGGTGCCCTTCCAC	222	circRNA
circRNA_0227	Reverse	AGCCTTTCCTTCTTCGCAACC		
circRNA_0974	Forward	TGACAAGAAATGACTCTCACATGCT	210	circRNA
circRNA_0974	Reverse	GTATGTTGGACGGCATACATCCT		
ptc-miR156g	Forward	cgcgTTGACAGAAGATAGAGAGCAC		miRNA
NW_011500297.1_28413	Forward	TTTTCTTCCCCTGTTCTCTGCGTG		miRNA
NW_011500300.1_28478	Forward	cgcgAATGAGAACCATCTGACATCC		miRNA
ptc-miR473a-3p	Forward	TGAGGCCTTTGGGGGAGAGTGG		miRNA
ptc-miR396c	Forward	cgcgTTCCACAGCTTTCTTGAACTT		miRNA
ptc-miR319e	Forward	gTTGGACTGAAGGGAGCTCCT		miRNA
XM_011034944.1	Forward	AGAGCGGAACTGGGTCATCCTC	85	mRNA
XM_011034944.1	Reverse	CTTGGTGGCTGCTGGTGCTG		
XM_011031202.1	Forward	TGCGAGGTTCATGCGAAGTCAC	138	mRNA
XM_011031202.1	Reverse	GTGTCCTGCCAAACGCCTACG		
XM_011022862.1	Forward	ACACCTCCTCCTCCTCCTCCTC	85	mRNA
XM_011022862.1	Reverse	GGTTCTTGGATAGCGGGTTCGG		
XM_011016288.1	Forward	GGAACGCCGATTTTGCCAACAG	110	mRNA
XM_011016288.1	Reverse	TCCGTCGCCTTGCATTGTGATC		
XM_011020593.1	Forward	ACCTGCCTCTCCGTTTCAATGC	97	mRNA
XM_011020593.1	Reverse	GCTCCGGCTCCTCCTCTGC		
XM_011037142.1	Forward	AGCCAGAGCCAGAACCAGAACC	109	mRNA
XM_011037142.1	Reverse	GAGCGAGCGACAACCCTGAAC		
XM_011049364.1	Forward	GCTAAGGCTCAGGTTGTGCT	112	mRNA
XM_011049364.1	Reverse	GCCTGCGACAGCTTCTTTTG		

RNAs* include mRNA, miRNA and circRNA.

Table S4 The validation of regulatory relationships between miRNAs, TF mRNAs and circRNAs.

RNAs*	Li1	Li2	Li3	Li4	Mean value	La1	La2	La3	La4	Mean value	Ov1	Ov2	Ov3	Ov4	Mean value	Bo1	Bo2	Bo3	Bo4	Mean value
circRNA_0168	1.01173E-07	9.66046E-08	9.50547E-08	9.75015E-08	9.75835E-08	2.5076E-05	2.4788E-05	2.11351E-05	1.95383E-05	2.26343E-05	7.90614E-05	8.14722E-05	8.16606E-05	8.83343E-05	8.26321E-05	9.22985E-05	9.96111E-05	8.95674E-05	0.000101939	9.58541E-05
circRNA_0958	6.27738E-07	5.9525E-07	5.81655E-07	5.63141E-07	5.91946E-07	8.01651E-05	7.6722E-05	6.42178E-05	6.33337E-05	7.11097E-05	4.7998E-05	4.60427E-05	4.70102E-05	5.5008E-05	4.90147E-05	6.85096E-05	7.05986E-05	6.64825E-05	7.60162E-05	7.04017E-05
circRNA_1102	2.80272E-07	2.73239E-07	2.609E-07	2.64542E-07	2.69738E-07	4.83318E-05	4.8109E-05	3.87172E-05	3.75715E-05	4.31824E-05	5.33803E-05	5.0268E-05	5.40005E-05	6.39218E-05	5.53926E-05	0.000274039	0.000288995	0.000265316	0.000301965	0.000282579
circRNA_0801	2.11634E-06	2.1608E-06	2.03482E-06	1.93845E-06	2.0626E-06	1.1031E-06	1.10056E-06	8.93931E-07	8.81624E-07	9.94804E-07	5.25429E-07	5.30308E-07	5.71002E-07	5.83E-07	5.52435E-07	5.89774E-07	5.17E-07	5.73647E-07	6.99745E-07	5.95042E-07
circRNA_0227	9.59373E-08	9.93205E-08	9.48353E-08	8.78733E-08	9.44916E-08	1.95716E-08	1.98907E-08	1.60446E-08	1.5897E-08	1.7851E-08	3.64375E-08	4.20496E-08	3.65218E-08	3.85151E-08	3.8381E-08	1.71503E-06	1.61131E-06	1.66044E-06	1.85091E-06	1.70942E-06
circRNA_0974	0.000184171	0.000183746	0.000183746	0.000188041	0.000184926	6.45153E-05	5.96411E-05	5.13242E-05	5.05008E-05	5.64954E-05	6.43664E-05	6.51143E-05	6.21738E-05	7.34268E-05	6.62703E-05	8.43453E-05	7.56658E-05	8.85386E-05	8.10965E-05	8.24116E-05
XM_011034999.1	0.001760255	0.001818124	0.00188224	0.001917355	0.001844494	0.000289664	0.000299187	0.000238014	0.000217505	0.000261092	0.000286999	0.000291678	0.000287663	0.000319182	0.000296381	0.000518513	0.000487154	0.000504334	0.000615196	0.000531299
XM_011031202.1	0.00271155	0.002717822	0.002595089	0.002404579	0.00260726	0.001744062	0.001692455	0.001497391	0.001497391	0.001607825	0.001443046	0.001466575	0.001397115	0.001724029	0.001507691	0.002939935	0.002912889	0.002846359	0.003107564	0.002951687
XM_011022862.1	0.000236917	0.000229376	0.000221051	0.000221562	0.000227226	0.001604864	0.001504326	0.001267909	0.001221893	0.001399748	0.002483631	0.002518301	0.002387969	0.002781348	0.002542812	0.002285407	0.002301303	0.002212664	0.002472181	0.002317889
XM_011016288.1	0.006375113	0.005989564	0.006360401	0.006316466	0.006260386	0.007494251	0.007155823	0.006272835	0.006419456	0.006835591	0.011979128	0.011491147	0.011951482	0.01257469	0.011999112	0.015268125	0.016101438	0.014816344	0.016901948	0.015771964
XM_011020593.1	0.009551877	0.009843133	0.009573972	0.009775142	0.009686031	0.009888723	0.009662865	0.007848685	0.007563834	0.008741027	0.009551877	0.009843133	0.009685216	0.011280697	0.010090231	0.010356402	0.009957505	0.010003625	0.01064756	0.010241273
XM_011037142.1	0.009269239	0.00912053	0.008668512	0.009205212	0.009065873	0.004656086	0.004613253	0.003636249	0.003670011	0.004143899	0.004157696	0.004128977	0.004374498	0.005095125	0.004439074	0.00383471	0.004044004	0.003712654	0.004528765	0.004030033
XM_011049364.1	1.01174E-07	9.62046E-08	9.52547E-08	9.74015E-08	9.75E-08	2.5176E-05	2.4988E-05	2.21351E-05	1.93383E-05	2.29E-05	7.95614E-05	8.24722E-05	8.26606E-05	8.85343E-05	8.33E-05	9.25985E-05	9.96311E-05	8.95686E-05	0.000201939	1.21E-04
ptc-miR156g	0.000440064	0.00044931	0.00044931	0.000427042	0.000441432	0.000229906	0.000231505	0.000194672	0.000187174	0.000210814	0.000186311	0.000181216	0.000182477	0.00021451	0.000191129	0.00019648	0.000184597	0.000190226	0.000202471	0.000193443
NW_011500297.1_28413	9.51129E-05	9.51129E-05	9.46744E-05	9.53329E-05	9.50582E-05	2.73772E-05	2.71881E-05	2.31815E-05	2.32889E-05	2.52589E-05	8.27199E-06	8.06441E-06	8.31031E-06	9.32801E-06	8.49368E-06	8.76387E-06	8.23386E-06	9.17835E-06	9.07293E-06	8.81225E-06
NW_011500300.1_28478	0.001334024	0.001358911	0.001321751	0.001210652	0.001306335	0.00068894	0.000705044	0.000583357	0.000579327	0.000639167	0.000445177	0.000437024	0.000433004	0.000499694	0.000453725	9.6218E-05	9.68873E-05	9.3371E-05	0.000104081	9.76394E-05
ptc-miR473a-3p	0.025976184	0.026583349	0.026217368	0.027016788	0.026448422	0.01332242	0.013508394	0.011306791	0.011306791	0.012361099	0.017742257	0.01551707	0.017701311	0.018115059	0.017268924	0.014816344	0.0149194	0.01434475	0.017177533	0.015314507
ptc-miR396c	0.000496243	0.000474929	0.000454531	0.000437024	0.000465682	0.001410087	0.001390674	0.001155981	0.001142703	0.001274861	0.00227487	0.00226962	0.00227487	0.002489376	0.002327184	0.002724109	0.002619184	0.002649618	0.002946736	0.002734912
ptc-miR3	0.02134	0.02042	0.02005	0.02080	0.02065	0.06871	0.07162	0.06065	0.05995	0.06523	0.06636	0.06792	0.06698	0.07484	0.06902	0.04161	0.04491	0.04029	0.04288	0.04242

19e	4379	7644	353	8739	8573	0132	796	0639	4007	5685	9613	0929	5841	2419	9701	7477	4757	2822	6478	7884

Mean value represented the fold changes between the randomly selected experimental group genes and 18S RNA as the control gene by three technical replicates and four biological replicates.

Table S5 qPCR validation of the regulatory relationship of circRNAs-miRNAs-TF mRNAs in *Populus euphratica* Oliv. heteromorphic leaves

**Correlation coefficien1(CC1)**	**circRNAs**	**miRNAs**	**TF mRNAs**	**Correlation coefficien2(CC2)**
-0.5255	circRNA_1102	ptc-miR156g	XM_0110349**44**.1	0.3545
-0.5255	circRNA_1102	ptc-miR156g	XM_011031202.1	-0.6504
-0.5255	circRNA_1102	ptc-miR156g	XM_011049364.1	-0.7278
-0.8399	circRNA_0168	NW_011500297.1_28413	XM_011031202.1	-0.5904
-0.8399	circRNA_0168	NW_011500297.1_28413	XM_011049364.1	-0.6039
-0.7703	circRNA_0168	ptc-miR156g	XM_011031202.1	0.382
-0.7703	circRNA_0168	ptc-miR156g	XM_011034944.1	-0.3545
-0.7703	circRNA_016**8**	ptc-miR156g	XM_011049364.1	-0.7278
-0.3948	circRNA_0168	ptc-miR39**6c**	XM_0110349**44**.1	-0.2904
-0.3948	circRNA_0168	ptc-miR39**6c**	XM_011031202.1	-0.3461
-0.3948	circRNA_0168	ptc-miR39**6c**	XM_011049364.1	-0.5628
-0.9124	circRNA_0958	NW_011500300.1_28478	XM_011022862.1	-0.7576
0.6515	circRNA_0227	ptc-miR396c	XM_011020593.1	0.5914
-0.3253	circRNA_0227	ptc-miR156g	XM_0110349**44**.1	-0.3427
-0.4411	circRNA_0801	ptc-miR473a-3p	XM_011016288.1	-0.4634
0.9769	circRNA_080**1**	ptc-miR156g	XM_011016288.1	-0.633
-0.5255	circRNA_1102	ptc-miR156g	XM_011031202.1	0.382
-0.0214	circRNA_1102	ptc-miR319e	XM_011034999.1	-0.9221
-0.9202	circRNA_0974	ptc-miR396c	XM_011034999.1	-0.7148
0.9717	circRNA_0974	ptc-miR156g	XM_011016288.1	-0.633
-0.9202	circRNA_0974	ptc-miR319e	XM_011037142.1	0.8202

CC1 and CC2 represent the correlation coefficient between circRNAs and miRNAs, TF mRNAs and miRNAs, respectively. The red represents the positive correlation coefficient.
